# Repair of mitral paravalvular leak using left atrial appendage tissue

**DOI:** 10.1093/icvts/ivae161

**Published:** 2024-09-28

**Authors:** Ryohei Otsuka, Shunei Saito, Tsukasa Ohno, Ken Miyahara

**Affiliations:** Department of Cardiovascular Surgery, Ichinomiya Municipal Hospital, Ichinomiya, Japan; Department of Cardiovascular Surgery, Ichinomiya Municipal Hospital, Ichinomiya, Japan; Department of Cardiovascular Surgery, Ichinomiya Municipal Hospital, Ichinomiya, Japan; Department of Cardiovascular Surgery, Ichinomiya Municipal Hospital, Ichinomiya, Japan

**Keywords:** Perivalvular leak, Paraprosthetic leak, Paravalvular leak, Repair, Left atrial appendage

## Abstract

Paravalvular leak after mitral valve replacement causes serious symptoms such as heart failure and haemolysis. However, whether re-replacement or direct leak site repair is the appropriate surgical treatment for this condition remains controversial. Herein, we describe a case of paravalvular leak repaired using left atrial appendage tissue with excellent results. The proposed technique enables the repair of a leak at the 9 o’clock position with healthy, full-thickness autologous tissue. For this method, the leak must be located near the left atrial appendage, and the left atrial appendage must not adhere to the pericardial sac. Although this technique can only be used under specific conditions, it is a useful option for cardiac surgeons.

## INTRODUCTION

Paravalvular leak after mitral valve replacement occurs in 7–17% of patients and causes serious symptoms such as heart failure and haemolysis. However, whether re-replacement or direct leak site repair is the appropriate surgical treatment for this condition remains controversial. Herein, we describe a case of paravalvular leak repaired using left atrial appendage tissue with excellent results.

## CASE REPORT

A 72-year-old man underwent mitral valve replacement (SJM Mechanical Valve #31, St Jude Medical, MN, USA) 17 years ago for mitral stenosis. He presented to our hospital with a chief complaint of shortness of breath on exertion. Blood tests showed lactate dehydrogenase (LDH) of 1904 U/l, B-type natriuretic peptide (BNP) of 222.5 pg/ml and haemoglobin of 8.1 g/dl. Echocardiography showed an ejection fraction of 55% and moderate to severe paravalvular regurgitation near the left atrial appendage (Fig. 1A). Electrocardiography detected atrial fibrillation/flutter with a pulse rate of 57 beats per minute. The patient was referred to our department for reoperation.

**Figure:**
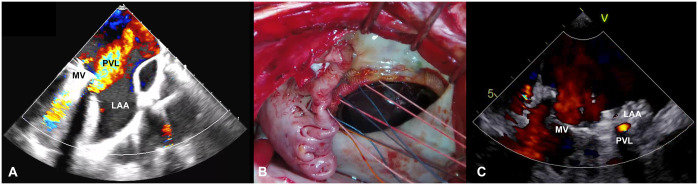
Figure1: (**A**) Preoperative echocardiogram. (**B**) An operative picture indicating the technique. (**C**) Postoperative echocardiogram. LAA: left atrial appendage; MV: mechanical valve; PVL: paravalvular leak.

**Figure 2:**
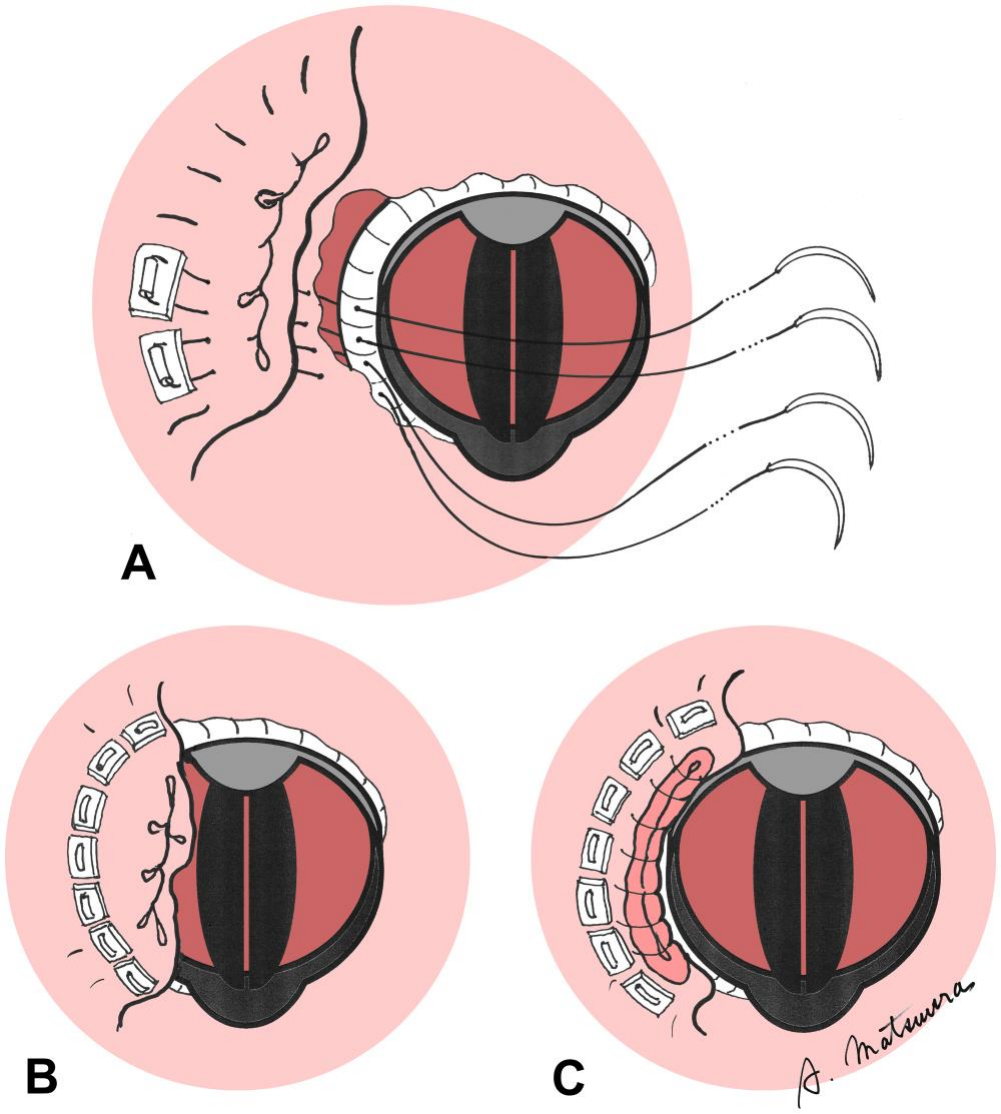
(**A**) and (**B**) The left atrial appendage was turned inward, and its tissue was sutured with 7 pairs of felted U-shaped sutures pressed against the sewing cuff. (**C**) The excess left atrial appendage tip was resected and closed in 2 layers.

Extracorporeal circulation was established through the femoral artery and vein, and the chest was then reopened. Sparse adhesions were dissected circumferentially. The aorta was clamped, and a right-sided left atrial incision was made. Dehiscence measuring ∼1 cm was recognized at the 9 o’clock position. The left atrial appendage was turned inward, and its tissue was sutured with 7 pairs of felted U-shaped sutures pressed against the sewing cuff. The excess left atrial appendage tip was resected and closed in 2 layers (Figs. 1B and 2, Video 1).

The patient was weaned off the ventilator on day 1 and left the intensive care unit on day 3. Postoperative echocardiogram showed trace residual perivalvular regurgitation (Fig. 1C). Moreover, postoperative LDH (359 U/l), BNP (22.5 pg/ml) and haemoglobin levels (10.0 mg/dl) improved compared with preoperative data. The patient was discharged home on day 16.

## DISCUSSION

Despite numerous reports of transcatheter paravalvular leak repair in recent years, the European and American guidelines still recommend operative treatment as the 1st option when the operative risk is reasonable. However, whether the procedure would be re-replacement or direct leak site repair remains controversial. According to Choi *et al.* [1], re-replacement is a risk factor for re-leakage compared with leak site repair. We have also experienced recurrent leaks after re-replacement; thus, leak site repair is now our 1st choice when possible.

In addition to direct suturing, xenogeneic pericardium has been reported to repair leaks in the mitral annulus. Kotani *et al*. [2] proposed the double-closure technique, in which the leak is closed directly with a pledgeted U-shaped suture and then reinforced with a pericardial patch. Ryomoto *et al*. [3] reported that bringing the left atrial tissue itself to the swing cuff is sometimes difficult, and they used a pericardial roll to fill this gap.

On the contrary, methods using autologous tissue have been reported: using atrial septum tissue from the right atrial side for a leak at the 12 o’clock position [4], using the lateral left atrial wall through the coronary sinus for a leak at the 3 o’clock position [5] and using the posterior left atrial wall from the epicardial side for a leak at the 6 o’clock position [4]. They claim that durability is improved by using healthy full-thickness autologous tissue. The proposed technique enables the repair of a leak at the 9 o’clock position with healthy full-thickness autologous tissue. For this method, the leak must be located near the left atrial appendage, and the left atrial appendage must not adhere to the pericardial sac. Needles should not go too deep in order not to damage the circumflex coronary artery. In the present case, heart failure and haemolysis were corrected after extensive repair using the left atrial appendage tissue and 7 pairs of U stitches. Although this technique can only be used under specific conditions, it is a useful option for cardiac surgeons.

## Data Availability

All relevant data are within the manuscript.
